# Vitality of Proteinase K in rRTPCR Detection of SARS-CoV2 Bypassing RNA Extraction

**DOI:** 10.3389/fcimb.2021.717068

**Published:** 2021-11-03

**Authors:** Alka Shukla, Mayank Gangwar, Gaurav Sharma, Pradyot Prakash, Gopal Nath

**Affiliations:** ^1^ Viral Research and Diagnostic Laboratory, Department of Microbiology, Faculty of Medicine, Institute of Medical Sciences, Banaras Hindu University, Varanasi, India; ^2^ Department of Public Health Dentistry, SriRama Chandra Bhanj Dental College & Hospital, Cuttack, India

**Keywords:** real time PCR, SARS-CoV-2 detection, heat inactivation, COVID – 19, Proteinase K

## Abstract

This study aimed to detect the SARS-COV2 viral component directly from inoculated VTM without RNA extraction. Inoculated VTMs of already tested 50 positive and 50 negative samples were divided into three groups. Group I was treated with Proteinase K (PK) followed by 3-step-heat treatment at different temperatures (25°C, 60°C, and 98°C) and stored at 4°C. Group II was directly subjected to 3-step-heat treatment without PK exposure and stored at 4°C. And group III was set-up as standard group; it was processed using Qiagen’s column based QIAamp Nucleic Acid kit and the obtained nucleic acids were stored at 4°C. These stored samples were used as a template to execute real-time polymerase chain reaction, and results were noted. Group I demonstrated 96% and 88% sensitivity for N and ORF1ab genes respectively, whereas group II demonstrated 78% and 60% when compared to the results of standard group III. Overall group I showed better results than group II when compared to group III. Thus, in situations where gold-standard reagents are not available, PK exposure and heat treatment can be employed to carry out molecular detection of SARS-CoV2 viral component.

## Introduction

In 1918, the entire globe was hit by Spanish flu which affected around 500 million people, which was approximately one-third of the entire world’s population (Frost, 1920). A century later, unfortunately, the world witnessed yet another horrible pandemic attack from coronavirus disease 19 (COVID-19) in the year 2019, which has lingered on for subsequent years. Up to 20^th^ May 2021, a total number of 164 million COVID-19 positive cases have been reported worldwide (Frost, 1920; Mohamadi et al., 2021). Timely diagnosis of the disease is the most important step to combat any pandemic disease. But, after this infectious respiratory disease bloomed, there had been exponentially increasing demand for diagnostic kits and reagents which led to an acute shortage of resources (Chaimayo et al., 2020); McMahon et al., 2020). Since the gold standard for COVID-19 diagnosis is real-time reverse-transcriptase polymerase chain reaction (rRTPCR) assay (Han and Yang, 2020), nucleic acid (NA) extraction is the mandatory requirement. NA extraction requires the employment of various reagents, column-based kits, sophisticated equipment, and trained laboratory personnel, along with time. These basic requirements of rRTPCR, in turn, resulted in delayed diagnosis of infected individuals and thus has drawn the attention of many researchers to curb the shortcomings. Automated NA extractors rose to prominence with all promising features to cut short on manpower and duration of NA extraction, and hence proved to be the perfect answer for all the aforementioned challenges faced during NA extraction. But this boon came with an expensive price that was not possible for all laboratories to afford. In India, many laboratories still perform manual NA extraction that delays reporting of infected positive cases.

Thus, in an attempt to address issues faced by average-budget diagnostic laboratories, the present study was designed to validate a simplified protocol which can be employed to obtain a template for COVID19 rRTPCR diagnosis by omitting the conventional NA extraction protocol.

For this purpose, proteinase K (PK), which possesses exceedingly superior protein-degrading properties and is widely used in NA extraction protocols, was brought into the picture (Yu et al., 2018). PK has been in use for a long time for NA extraction procedures. Moreover, owing to its lytic property, Yu F et al. have used it to homogenize viscous sputum of Influenza-A suspected samples prior to NA isolation (Yu et al., 2018). Although they had to use conventional ribonucleic acid (RNA) extraction protocol to obtain viral RNA, PK assisted in pre-treatment of mucus-sputum to make it desirable for NA extraction (Yu et al., 2018). However, apart from the above mentioned role, its direct application on inoculated viral transport media (VTM) to obtain NA by skipping conventional protocols has gained popularity in recent years (Mallmann et al., 2020; Michel et al., 2020; Genoud et al., 2021).

## Methodology

### Selection of Samples

This study was performed in the Department of Viral Research and Diagnostic Laboratory (VRDL), Department of Microbiology, Institute of Medical Sciences, Banaras Hindu University (IMS, BHU). This VRDL is a level II biosafety laboratory. Viral transport media (VTM), inoculated with nasopharyngeal and oro-pharyngeal swabs from COVID 19 suspected patients, have been tested in this lab since March 2020 till date on a routine basis. The study design was approved by the Institutional Ethics Committee and research board (Dean/2020/EC/887). However, individual signed informed consent was exempted due to the retrospective nature of the study. This lab is a DHR-Central Government approved COVID-19 testing laboratory. Only the routine COVID-19 samples received at the Indian Council of Medical Research (ICMR), New Delhi, India approved state level laboratory were selected for the present work. The routine testing of these samples is carried out following the standard protocol of NA extraction (using column-based QIAamp Viral RNA Kit) and subsequent rRTPCR assay. Out of the stored samples in VTM, 50 positive and 50 negative samples were included in this study. These samples were serially numbered from 1 to 100 and were divided into three groups as per protocol.

### Experimental Protocol of Proteinase K Treatment

**Figure 1:**
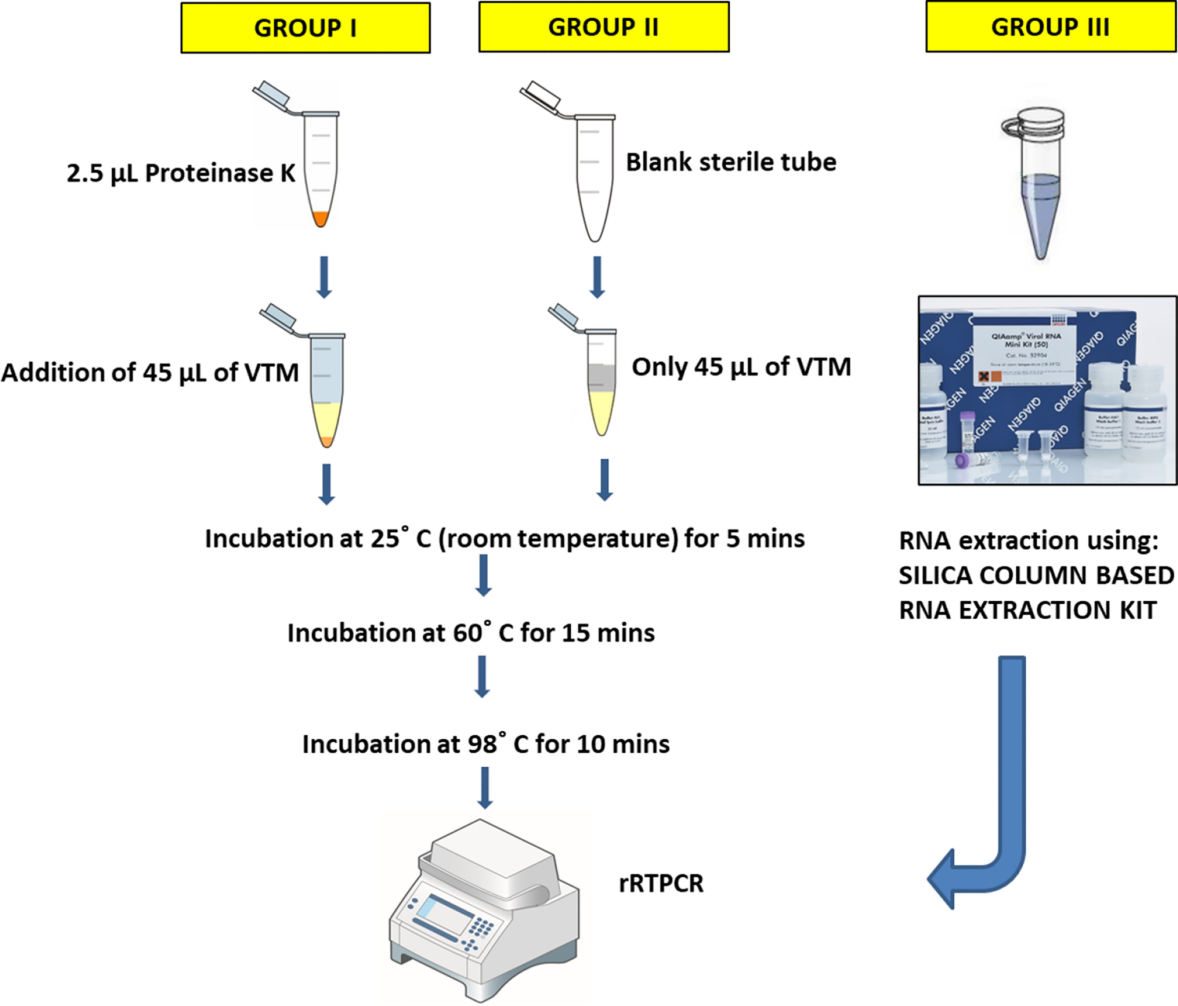
Figurative representation of experimental protocols. Group I protocol: VTM samples were treated with PK prior to 3-step-heat treatment; Group II protocol: VTM samples were directly subjected to 3-step-heat cycles to obtain template for rRTPCR; Group III: Standard WHO COVID19 RT-PCR protocol was employed, using column-based RNA extraction.

Two streamlined, trouble-free protocols involving shorter duration and lesser handling of the VTMs before molecular detection of concerned virus were designed. Handling of samples was done inside a Class II, Type A2 Biological safety cabinet. Two groups, group I and group II, of 45 µL of VTMs of each of the above mentioned 100 samples were formed. Group I included addition of 2.5 µL (20mg/ml concentrated) proteinase K (PK), making a final concentration of PK ~1 mg/ml VTM, whereas group II did not get any PK exposure. **Figure 1** represents a flow chart of experimental protocols followed. After heat treatment, these samples were stored at 4°C. 5 µL of this stored sample was used as a template to execute rRTPCR assay. Group III was the standard group which was set up to compare the results of experimental groups I and II. Group III consisted of aliquots from the same set of samples used in the previous two groups; however, group III samples were processed for NA extraction using Qiagen’s column based QIAamp Nucleic Acid kit. 5 µL of extracted RNA was used as template to execute rRTPCR assay. The RT-PCR kit employed for molecular detection was Q-Line^®^ Molecular ER nCOV-19 RT-PCR Kit which targeted two viral genes: ORF1ab and Nucleoprotein N genes. All the three groups were subjected to rRTPCR simultaneously in a BIORAD CFX96™ Real-Time Thermocycler. Once the rRTPCR run was over, negative and positive controls were checked for the validation of run and the C_t_ values were noted down. The C_t_ value obtained from experimental groups I and II were recorded, compared with group III, and subjected to statistical analysis to draw conclusions.

### Statistical Analysis

The data obtained was entered into a Microsoft Excel worksheet and analyzed using SPSS ver. 18. Mean and standard deviation were computed for continuous variables. One Way Analysis of Variance (ANOVA) was employed to compare continuous variables. Pairwise comparisons were done using *Post Hoc* Tukey’s test after Bonferroni adjustment for multiple comparisons. A *p*<0.05 was considered significant for all statistical inferences. Receiver operating characteristic (ROC) curve was plotted to assess diagnostic accuracy of ORF1ab and N gene.

## Results

**Figure 2:**
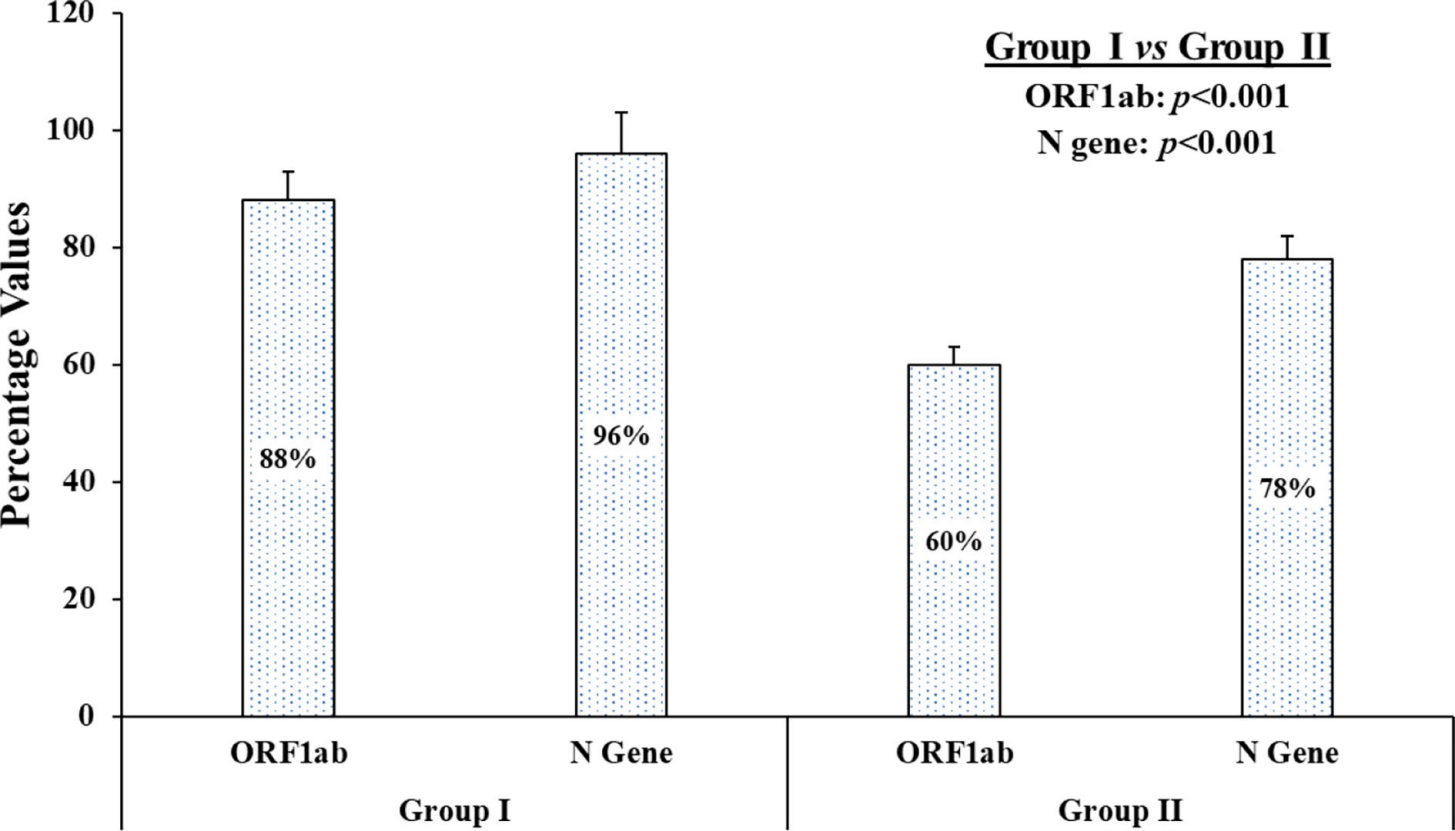
The graphical presentation of concordance results of known positive samples. On qualitative analysis of 50 known samples, the concordance percentage depicts individual genes, i.e., ORF1ab gene and N gene, of each experimental group (group I and group II) when compared to standard group (group III).

In group I and II, samples which showed threshold cycle (C_t_) values less than 40 for any of the two genes were considered positive as per manufacturer’s instructions. The obtained C_t_ values after treatment in group I and II, along with Ct values obtained in standard control group III, were tabulated in the supplementary data (**Table S1**). In terms of qualitative analysis of known 50 positive samples, i.e., whether the sample is positive or negative for the targeted genes (irrespective of its low or high Ct value), the following results were obtained: Group III (50 samples positive for ORF1ab and N gene), group I (44 samples positive for ORF1ab and 48 for N gene), and group II (30 samples positive for ORF1ab and 39 for N gene) Although results of both the experimental groups (I and II) for both the genes deviated from the standard group (group III), group I showed greater concordance in terms of qualitative analysis than group II (**Figure 2**). Also, a significant difference was found between the concordance percentage for both the individual genes, *i.e.*, N gene (*p*<0.001) and ORF1ab gene (*p*<0.001), among the experimental groups respectively (**Figure 2**).

**Figure 3:**
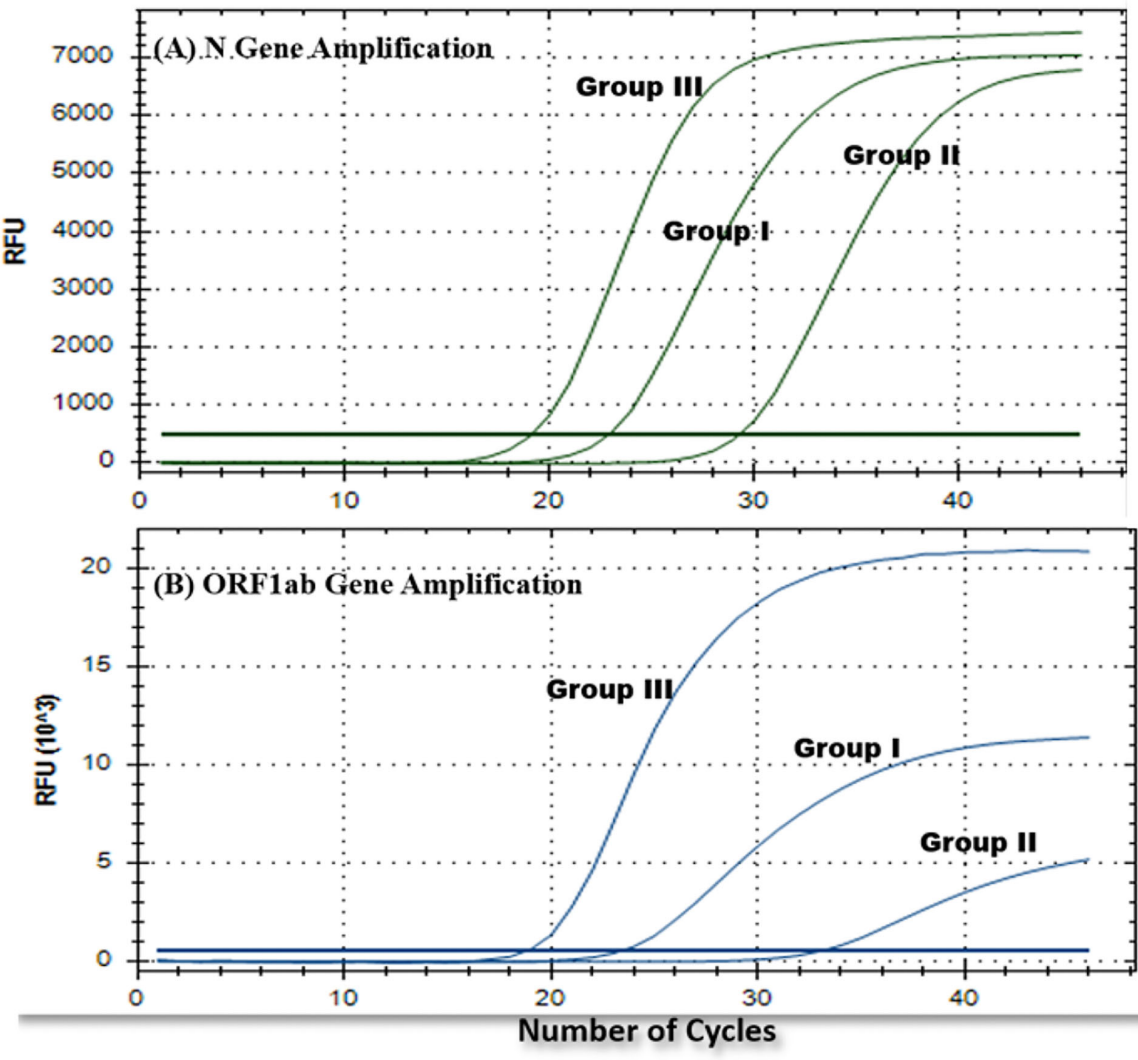
Representative rRTPCR amplification graphs for each gene **(A)** N gene and **(B)** ORF1ab gene, obtained for one of the common positive samples from each experimental group (group I, II, and III).

Amplication curves of group I and group II clearly showed delayed rise and decreased Relative fluorescence units (RFUs) for both the genes when compared to original amplication graphs of group III (**Figure 3**). Mean C_t_ values of ORF1ab and N gene were calculated separately. In comparision with reference group III, mean C_t_ values of ORF1ab gene for group I and II increased by 5.43 and 7.81, and for N gene it increased by 5.19 and 6.95 respectivly (**Table 1**). Stastistically significant difference in mean C_t_ values of both the targeted genes among all three groups can be well appreciated (**Table 2**).

**Table 1:** ANOVA for ORF1ab gene and N gene.

Variable	Group	Mean C_t_ value	Standard error	95% confidence interval	F	*p*
ORF1ab	I	28.55 ± 5.13	0.774	26.98-30.11	30.16	<0.001
	II	30.93 ± 4.58	0.837	29.22-32.65		
	III	23.12 ± 4.29	0.608	21.9-24.34		
N gene	I	28.83 ± 4.75	0.687	26.95-29.72	29.43	<0.001
	II	30.59 ± 4.45	0.714	29.14-32.04		
	III	23.64 ± 3.99	0.565	22.51-24.77		

**Table 2:** *Post hoc* test for ORF1ab gene and N gene.

Variable	Group	Comparison	Mean Difference	*p*	95% Confidence Interval
ORF1ab	I	II	-2.38	0.01	-4.58 – -0.19
		III	5.43	0.001	3.51 – 7.34
	II	III	7.81	0.001	5.67 – 9.95
N gene	I	II	-1.76	0.01	-4.14 – -0.38
		III	5.19	0.001	2.93 – 6.45
	II	III	6.95	0.001	5.09 – 8.81

**Figure 4:**
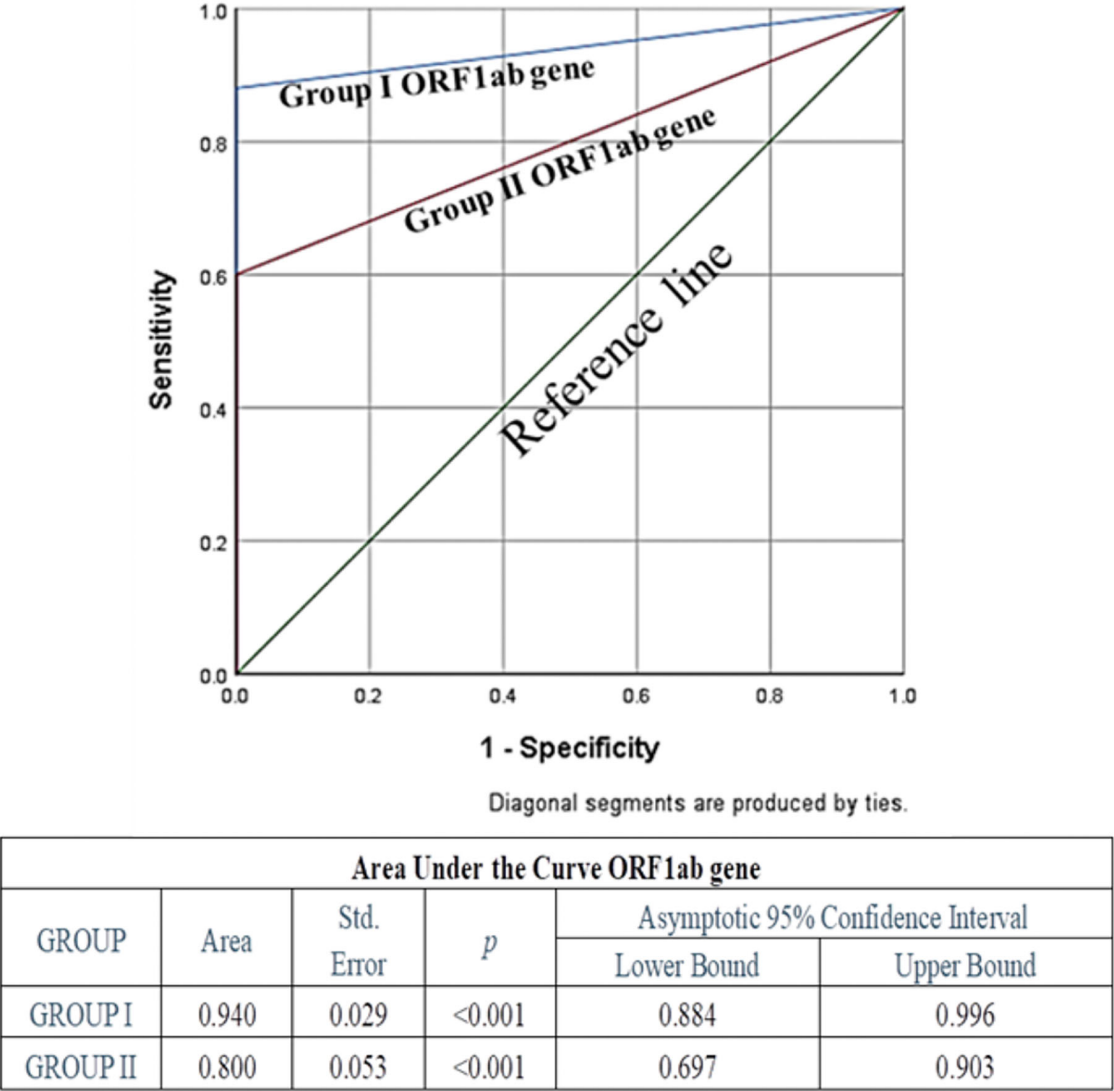
Receiver Operating Characteristic (ROC) Curve of ORF1ab gene to assess diagnostic accuracy showed excellent and significant area under the curve 0.94 (p<0.001) for Group I, and fair and significant area under the curve 0.8 (p<0.001) for Group II.

**Figure 5:**
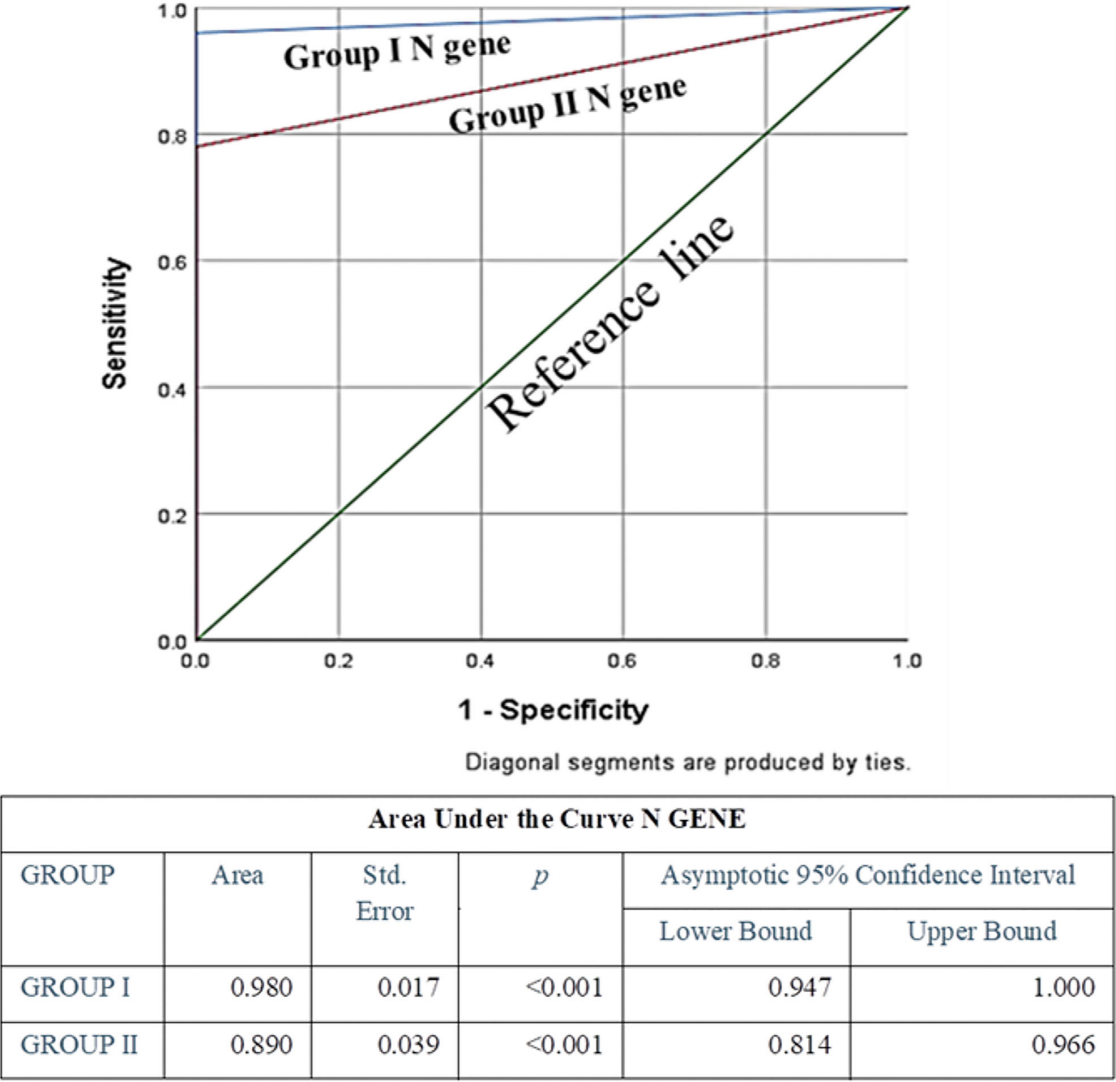
ROC Curve of N gene to assess diagnostic accuracy showed excellent and significant area under the curve 0.98 (p<0.001) for Group I, and excellent and significant area under the curve 0.89 (p<0.001) for Group II. Area under the curve was the maximum for N Gene in Group I, indicating its excellence in diagnostic accuracy.

Specificity of both the genes in both the groups was the same (100%), whereas sensitivity and diagnostic accuracy of both the genes for group I were found to be more promising than group II; among these two genes, N gene was found to be more sensitive and accurate than ORF1ab. Also, false negativity of group I for both the genes was noticeably less in comparison to group II (**Table 3**). None of the known negative samples for SARS-CoV2 were detected as positive in either of the experimental protocols of this study. Receiver Operating characteristic curve was used to choose the most appropriate cut-off for test and compare the usefulness of groups for either gene, where a greater area means more useful test (**Figures 4** and **5**).

**Table 3:** Various diagnostic parameters of ORF1ab and N genes of group I and II.

Group		Sensitivity (95% CI)	Specificity (95% CI)	False Negative	Negative Predictive Value (95% CI)	Diagnostic Accuracy (95% CI)
**ORF1ab gene**	**Group I**	88% (75.68- 95.47%)	100% (65.09- 97.09%)	12%	72.73% (55.73 - 84.96%)	90.91% (81.26 - 96.59%)
	**Group II**	60% (45.18-73.59%)	100% (79.41-100%)	20%	44.44% (36.29-52.91%)	69.70% (57.15-80.41%)
**N gene**	**Group I**	96% (86.29-99.51%)	100% (79.41-100%)	4%	88.89% (67.29-96.89%)	96.97% (89.48-99.63%)
	**Group II**	78% (64.04-88.47%)	100% (79.41-100%)	22%	59.26% (46.23-71.03%)	83.33% (72.13-91.38%)

CI, Confidence interval.

## Discussion

In this current pandemic, a shortage of reagents might lead to failure in on-time testing and screening of suspected samples (McMahon et al., 2020; Habli et al., 2021). Thus, in order to bridge the gap between available resources and demand of testing and also to reduce testing timing, new options are being explored by researchers across the globe. Since NA extraction is one of the main steps in whole rRTPCR based diagnostic procedure, several attempts have been made to replace chemical/kit-based standard NA extraction protocol, but none of these studies have reported to be 100% sensitive (Mallmann et al., 2020; Michel et al., 2020; Genoud et al., 2021). Mallmann L et al., had experimented with various strategies by subjecting the pure samples and phosphate-buffered saline (PBS) diluted sample to different thermal cycles of 70°C and 98°C for 5 and 8 minutes respectively (Mallmann et al., 2020), whereas in our study only pure undiluted inoculated VTM samples were used and were subjected to room temperature, i.e. 25°C, for 5 minutes followed by 60°C for 15 minutes and 98°C for 10 minutes. Genoud et al., reported 99% sensitivity by using PK and heat inactivation method of saline-inoculated samples in extraction-free protocol (Genoud et al., 2021). However, no attempt was made in samples inoculated in VTM, which is the recommended media for COVID19-suspected sample collection guidelines by the Indian Council of Medical Research, National Institute of Epidemiology (Piras et al., 2020).

The present study was designed to obtain better results by using PK, as it is readily available in all molecular laboratories and has been proven to play a key role in NA extraction (Wiegers and Hilz, 1971a; Tsuji et al., 2017). PK is an enzyme which breaks down proteins, inactivates nucleases, and has shown maximum efficiency at 60°C (Yazawa et al., 2016 **)**. Thus, in our study the PK exposed-inoculated VTM was incubated at room temperature and subjected to 60°C for 15 minutes to attain higher efficiency of PK. Further, it was subjected to 98°C for 10 minutes to inactivate the virus and PK (Darnell et al., 2004; Michel et al., 2020 **)** and later stored at 4°C till it was used as template to execute rRTPCR assay. There was an increase in the C_t_ values of both experimental groups. Also, the amplification graphs demonstrated a delayed peak with lower RFUs (**Table 1** and **Figure 3**). These results clearly indicate that the proposed protocols are not as sensitive and reliable as gold standard nucleic acid extraction protocol and thus should be used only in adverse conditions where it is difficult or impossible to execute standard protocol.

But it is intriguing to observe 100% specificity by using the PK addition in the sample. On comparison of group I and group II, a significant difference in qualitative as well as quantitative terms was observed. Mean Ct values for both the genes were lower and amplification graphs showed early peaks with higher RFUs for group I when compared to group II. These findings compel us to acknowledge PK treatment as a pivotal step for molecular detection of the viral component from VTM. The sensitivity of group I, which was far superior to group II (**Table 3**), can be attributed to PK-exposure (Wiegers and Hilz, 1971b **)**. False negativity (FN) detected in group I was 2%, which could be due to possible impurities present in inoculated VTM that caused a hindrance in rRTPCR run or degradation of NA due to heating (Fomsgaard Anna and Rosenstierne, 2020). In group II FN was 22%, which can be attributed to the presence of nuclease enzymes resulting in lytic degradation of NA (Yang, 2011). Apart from that, the time required to obtain the template with this technique for 24 samples was 30 minutes. On the other hand, time duration for standard column-based RNA extraction method for roughly 24 samples is approx. 1.5 hours (Genoud et al., 2021). In addition to that, column-based RNA extraction method requires more manpower and involves tedious mechanical steps. Whereas, by employing direct inoculated VTM technique, there is no involvement of sophisticated equipment. It requires only one water bath to obtain 0-100°C temperature and can be conveniently performed by a trained and experienced lab technician following proper biosafety measures.

Thus, taking all the parameters into consideration, we suggest PK exposure and heat treatment can be employed to screen samples in urgent situations, such as a pandemic, when there is a scarcity of resources. Further, it was evident from **Table 1** that Ct values had increased for the experimental-protocol groups I and II when compared to standard-protocol group III. Thus, it may be suggested that cut off C_t_ values should be fixed at a relatively higher level when employing these experimental protocols. Further studies are recommended to obtain more sensitive and accurate techniques.

## Data Availability Statement

The raw data supporting the conclusions of this article will be made available by the authors, without undue reservation.

## Ethics Statement

The studies involving human participants were reviewed and approved by Dean, Institute of Medical Sciences, and Banaras Hindu University. Written informed consent for participation was not required for this study in accordance with the national legislation and the institutional requirements.

## Author Contributions

AS, MG, and GN designed the experiment. AS and MG performed modified experimental procedures, detailed testing methodology. GS statistically analysed and interpreted the data significance. PP, AS, and GN reviewed, modified and finalized the final data and text representation. All authors contributed to the article and approved the submitted version.

## Conflict of Interest

The authors declare that the research was conducted in the absence of any commercial or financial relationships that could be construed as a potential conflict of interest.

## Publisher’s Note

All claims expressed in this article are solely those of the authors and do not necessarily represent those of their affiliated organizations, or those of the publisher, the editors and the reviewers. Any product that may be evaluated in this article, or claim that may be made by its manufacturer, is not guaranteed or endorsed by the publisher.
