# Supplementary material for: Vitality of Proteinase K in rRTPCR Detection of SARS-CoV2 Bypassing RNA Extraction
Source: Front Cell Infect Microbiol. 2021 Nov 3;11:717068. doi: 10.3389/fcimb.2021.717068 (PMC8595283; doi:10.3389/fcimb.2021.717068)
Supplement: Supplementary file 1 [file Table_1.docx]

Table S1: Ct values (N and ORF1ab gene) of the samples of all the three groups

| **Sample No.** | **N Gene** | | | **ORF1ab gene** | | |
| --- | --- | --- | --- | --- | --- | --- |
|  | **Group I** | **Group II** | **Group III** | **Group I** | **Group II** | **Group III** |
|  | 32 | 35 | 27 | 32 | NA | 27 |
|  | 32 | NA | 29 | 31 | NA | 30 |
|  | 36 | NA | 27 | 34 | NA | 27 |
|  | 36 | 36 | 29 | 35 | NA | 29 |
|  | 34 | 33 | 29 | 33 | 32 | 30 |
|  | 21 | 24 | 27 | 20 | 24 | 28 |
|  | 21 | 24 | 17 | 20 | 24 | 19 |
|  | 28 | 27 | 23 | 28 | 26 | 25 |
|  | 33 | 33 | 27 | NA | NA | 26 |
|  | 33 | 35 | 28 | NA | NA | 27 |
|  | 31 | 34 | 26 | 34 | NA | 25 |
|  | 21 | 26 | 19 | 21 | 28 | 17 |
|  | 32 | NA | 29 | 36 | NA | 29 |
|  | 30 | NA | 27 | 35 | NA | 26 |
|  | 28 | 31 | 28 | 30 | 34 | 27 |
|  | 22 | 23 | 22 | 23 | 24 | 21 |
|  | 21 | 22 | 22 | 22 | 24 | 20 |
|  | 26 | 28 | 26 | 28 | 30 | 25 |
|  | 23 | 26 | 23 | 24 | 28 | 24 |
|  | 33 | NA | 26 | NA | NA | 25 |
|  | 20 | 22 | 18 | 20 | 22 | 17 |
|  | 30 | 39 | 24 | 32 | NA | 23 |
|  | 31 | NA | 27 | 32 | NA | 26 |
|  | NA | NA | 29 | 34 | NA | 30 |
|  | 26 | 32 | 23 | 27 | 34 | 23 |
|  | 25 | 33 | 22 | 26 | 37 | 21 |
|  | 22 | 26 | 18 | 20 | 26 | 16 |
|  | 34 | NA | 27 | 35 | NA | 26 |
|  | 27 | 31 | 19 | 27 | 31 | 18 |
|  | 30 | 33 | 24 | 30 | 33 | 23 |
|  | 29 | 33 | 22 | 28 | 34 | 20 |
|  | 30 | 34 | 25 | 31 | 35 | 24 |
|  | 30 | 34 | 25 | 30 | 35 | 25 |
|  | 24 | 30 | 21 | 23 | 31 | 20 |
|  | 22 | 25 | 19 | 22 | 30 | 18 |
|  | NA | NA | 21 | NA | NA | 21 |
|  | 33 | 32 | 28 | 37 | 37 | 27 |
|  | 27 | 31 | 17 | 31 | 39 | 17 |
|  | 23 | 29 | 20 | 23 | 33 | 20 |
|  | 30 | 36 | 27 | 31 | NA | 26 |
|  | 25 | 31 | 22 | 26 | 35 | 22 |
|  | 30 | 32 | 19 | 34 | 36 | 19 |
|  | 28 | 32 | 18 | 28 | 33 | 17 |
|  | 26 | 30 | 17 | 27 | 32 | 16 |
|  | 32 | 38 | 24 | 34 | NA | 24 |
|  | 33 | 36 | 24 | 34 | NA | 23 |
|  | 26 | 31 | 18 | 26 | 33 | 17 |
|  | 22 | 26 | 17 | 22 | 28 | 15 |
|  | 36 | NA | 28 | NA | NA | 28 |
|  | 36 | NA | 28 | NA | NA | 27 |

NA: No amplification
